# The Effects of Chronic Stress on Migraine Relevant Phenotypes in Male Mice

**DOI:** 10.3389/fncel.2018.00294

**Published:** 2018-09-19

**Authors:** Dan Kaufmann, K. C. Brennan

**Affiliations:** Headache Physiology Lab, Department of Neurology, University of Utah, Salt Lake City, UT, United States

**Keywords:** migraine, chronic variable stress, social defeat, cortical spreading depression, nitroglycerin test, open field, elevated plus maze, mechanical allodynia

## Abstract

Migraine is a disabling neurological disorder affecting 12% of the world’s population. Stress is a major reported trigger and exacerbator of migraine. We evaluated the effects of two chronic stress paradigms on migraine relevant phenotypes in male C57Bl/6 mice.

**Methods:** Fifty six mice were used in a 14 day social defeat stress (SDS) and twenty three mice were used in a 40 day chronic variable stress (CVS) paradigm. Anxiety measures were evaluated using the open field and elevated plus maze (EPM) tests. Migraine relevant phenotypes were evaluated using the nitroglycerin (NTG) and cortical spreading depression (CSD) models.

**Results:** Stress sensitive SDS mice and chronically stressed CVS mice showed decreased exploration in the open field and reduced time spent in the open arms of the EPM compared to controls. Stress sensitive and resilient SDS mice had increased serum corticosterone levels, and stressed mice in the CVS paradigm had decreased weight gain compared to controls, providing combined behavioral and physiological evidence of a stress response. In the CVS paradigm but not the SDS paradigm, the stressed group showed a significant decrease in baseline mechanical withdrawal threshold compared to controls. All groups showed a significant reduction in withdrawal threshold after treatment with NTG, but the reduction was not larger in SDS or CVS than in controls. Interestingly, stress resilient SDS mice showed a rapid recovery from NTG effects that was not seen in other groups. No difference in CSD frequency or velocity was seen between stress and control mice in either stress paradigms.

**Conclusion:** We observed distinct effects of stress on generalized pain response, migraine relevant pain, and migraine relevant excitability. CVS but not SDS was associated with a reduced mechanical withdrawal threshold, consistent with a generalized pain response to chronic stress. Neither SDS nor CVS exacerbated phenotypes considered specifically relevant to migraine - withdrawal to NTG, and susceptibility to CSD. However, the significantly reduced response of stress resilient mice to the NTG stimulus may represent a specific migraine-resistant phenotype.

## Introduction

Exposure to stressors results in changes to both physiology and behavior which help an organism better adapt to external or internal changes (McEwen, 2013; Sapolsky, 2015). Though acute stress is adaptive, chronic unremitting stress can result in neuroanatomical, physiological and endocrine changes which underlie mood disorders like anxiety, depression, and PTSD (Drevets, 2000; de Kloet et al., 2005; McEwen, 2013). Stress also appears to have a profound effect on pain conditions, and depending on the nature of the stressor can induce both analgesia and hyperalgesia (Johnson and Greenwood-Van Meerveld, 2014; Burke et al., 2017; Li et al., 2017; Vachon-Presseau, 2017).

Migraine is a disabling neurological disorder affecting 12% of the world’s population (Buse et al., 2013) and imposes a high burden on sufferers, their families, and society at large. One of the most commonly reported triggers of migraine is stress (Rasmussen, 1993; Holm et al., 1997; Chabriat et al., 1999; Wober et al., 2006; Kelman, 2007). Stress is also an aggravating factor for migraine (Spierings et al., 2001; Nash and Thebarge, 2006) and is frequently cited as the most frequent factor in headache chronification (Nash and Thebarge, 2006; Rains, 2009; Borsook et al., 2012; May and Schulte, 2016).

In this work we evaluated the effects of two stress paradigms, social defeat stress (SDS) and chronic variable stress (CVS), on migraine relevant phenotypes in male mice. Our goal was to determine whether, and to what extent, stress affected two migraine models – the algesic response to nitroglycerin (NTG) which induces attacks in migraineurs but not patients without migraine (Thomsen et al., 1994; Iversen, 1995; Christiansen et al., 2000; Bates et al., 2010); and the susceptibility to cortical spreading depression (CSD), a wave of depolarization that is the physiological correlate of the migraine aura (Charles and Brennan, 2009; Brennan and Pietrobon, 2018). We reasoned that given the far-reaching effects of stress on network activity, either migraine-relevant pain (NTG) or cortical excitability (CSD) could be affected.

## Materials and Methods

All experiments, analysis, and reporting were performed according to ARRIVE criteria (Kilkenny et al., 2010). All experiments were approved by the Institutional Animal Care and Use Committee of the University of Utah.

**FIGURE 1:**
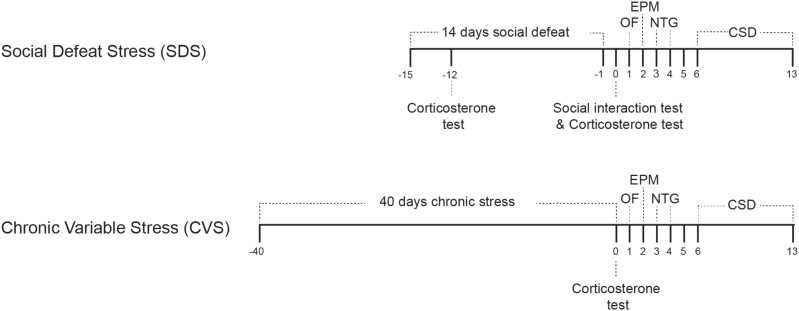
Experimental timeline for social defeat stress (14 days) and chronic variable stress (40 days) paradigms. Following either stress paradigm, Open field (OF) test was performed on day 1, elevated plus maze test on day 2. Mice were acclimated to the NTG apparatus on day 3 and mechanical withdrawal threshold was performed on day 4. CSD evaluation was performed for a week starting on day 6. ELISA corticosterone level evaluation was performed three days after initiation of the SDS paradigm, after the social interaction test, and on the last day of the CVS paradigm.

Experimental timelines are depicted in **Figure 1**. Following both paradigms we employed two behavioral tests, open field and elevated plus maze (EPM) tests, on days 1 and 2, to evaluate anxiety phenotypes. NTG test was done on days 3 (acclimation) and 4 (mechanical withdrawal threshold evaluation), and CSD testing was done during the week starting from day 6 (day 5 was reserved for rest after NTG injection) until day 13. The wider time range for CSD was because only 2 mice could be evaluated per day.

### Animals

Experiments were performed on C57Bl/6 male mice (6 weeks old; Jackson labs). Fifty six male mice were used for the SDS paradigm and twenty three male mice were used in the CVS paradigm. Mice were randomly divided into chronic stress and control groups (*n* = 28 per group in the SDS, and *n* = 12, 11 for the control and stress groups in the CVS). More mice were used for the SDS paradigm as SDS generates two groups: stress sensitive and stress resilient animals (Golden et al., 2011). Twenty eight retired CD-1 breeders (aggressor mice; Charles River Laboratories) were used in the SDS paradigm and were singly housed throughout the experiment. Fifty six additional animals were used for plasma corticosterone evaluation after both stress paradigms (six per group after 3 days and thirty two after 14 days for the SDS and six per group for the CVS paradigm). Animals were housed in clear acrylic cages (30 cm × 15 cm × 20 cm) and were allowed free access to food and water (Product 2920X, Harlan Teklad), except for chronically stressed mice which were denied food and water for no longer than 24 h during separated days of the chronic stress paradigm. Animals were kept in a temperature, humidity, and 12:12 h light:dark cycle controlled environment. Every attempt was made to minimize pain or distress experienced by experimental animals.

### Social Defeat Paradigm

Aggressor mice were singly housed and acclimated to their home cage for a week prior to screening. Initially, aggressor mice were screened for their aggressive behavior by placing three naïve C57Bl/6 mice, one at a time, for 3 min in the retired breeder’s home cage. This was repeated for three consecutive days. If an aggressor mouse failed to initiate an attack on 2 out of 3 mice, or if the latency for attack was greater than 1 min, they were excluded. Five or six aggressor mice were included in each trial of social defeat test. They were acclimated to one side of a large clear acrylic cage (35 cm × 15 cm × 25 cm) divided by a perforated clear acrylic divider. On each day of testing, six naïve C57Bl/6 mice were placed on the same side of the aggressor for 5 min during their light cycle. At the end of 5 min, naïve mice were placed on the opposite side of the divider for 24 h, so that both visual and scent cues of the aggressor were still present, but with no further physical attacks. The following day each mouse was exposed to a different aggressor to prevent habituation, so that all mice were rotated between all six aggressors for the length of the 14 day experiment. Control mice were housed in similar conditions as the social defeat mice and were handled daily using the cupped hand method (Hurst and West, 2010).

### Social Interaction Test

On day 15, C57Bl/6 mice were screened for stress sensitivity using the social interaction (SI) test. In this test, an opaque white rectangular open field arena (43 cm × 43 cm × 43 cm) having an enclosed wire mesh compartment (8 cm × 11 cm × 43 cm) was used. All mice were acclimated to the testing room for 1 h prior to initiation of the test. Each mouse was placed in the center of the open field arena, opposite to the wire mesh compartment, for 2 sessions of 5 min each, either with or without a novel aggressor present in the wire mesh compartment. The interaction zone with the aggressor was defined according to a previously published method (Golden et al., 2011). The total distance moved and time spent in the interaction zone were recorded in each session. A SI ratio was determined based on the time spent in the interaction zone when aggressor was present divided by the time spent in the interaction zone before placing an aggressor. Animals whose SI score was higher than 1 were considered stress resilient, and those with an SI score lower than 1 were considered stress sensitive (Golden et al., 2011).

### Chronic Variable Stress Paradigm

Seven different stressors were randomly deployed for 40 consecutive days (Gamaro et al., 2003, 2014) (**Supplementary Table 1**). Randomization prevented habituation of the animals to the stressors (Willner, 2017). The stressors employed were: rat encounter, predator odor, restrained stress, tail suspension, wet bedding, food and water deprivation, and cage tilt (Gamaro et al., 2003; Willner, 2017). Control mice were handled daily for 40 days during mid-morning using the cupped hand method (Hurst and West, 2010).

### Corticosterone ELISA Assay

Mice were anesthetized using isoflurane, and blood samples were obtained by cardiac puncture. Blood was collected in ice-chilled Microtainer blood collecting tubes (BD Life Sciences, Franklin lakes, NJ, United States) and centrifuged at 3000 G for 10 min to separate plasma from cellular components. Plasma samples were stored at -80°C until use. Plasma corticosterone levels were evaluated using ELISA according to the manufacturer’s instructions (Arbor Assays, Ann Arbor, MI, United States). All samples and standards were assayed in duplicate.

### Behavioral Evaluation of Anxiety

The behavior room was illuminated with red light in order to increase ambulatory activity of the mice during behavior testing (Robinson et al., 2018). Before commencement of behavioral tests, mice were allowed to acclimate to the behavior room for 1 h on the day of testing. Open field and EPM tests were performed in order, once daily for 2 days. 10–12 mice were tested per day from 9 am to 5:30 pm.

### Open Field Test

Each mouse was placed in a circular clear acrylic chamber (11 cm diameter × 7 cm height) located next to the wall of an illuminated (330 lux) circular open field (OF) arena (110 cm diameter) and allowed to acclimate for 1 min to decrease movement bias resulting from experimenter handling. After 1 min the chamber was manually removed from outside the arena, and the mouse was allowed to freely explore the OF arena for 30 min. Movement was video recorded and analyzed using Ethovision v.9 (Noldus, Leesburg, VA, United States). Data in the open field test was evaluated using the Software for the Exploration of Exploration (SEE) (Drai and Golani, 2001) which uses a set of statistical algorithms which divide the mouse movement into distinct ethologically relevant segments, and determines the center vs. periphery for each mouse individually, instead of having a predetermined center by the experimenter.

### Elevated Plus Maze

The EPM apparatus was elevated 60 cm from the floor, with two open arms (30 cm × 5 cm × 0.5 cm) and two closed arms (30 cm × 5 cm × 16 cm) connected by a central platform (5 cm × 5 cm) (Holmes et al., 2000; Walf and Frye, 2007). The EPM was illuminated by a white light (205 lux) at the center platform. Each mouse was placed in a rectangular opaque white acrylic chamber (5 cm × 7.5 cm × 12 cm) located on the center platform, and allowed to acclimate for 1 min before commencement of the test. The chamber was mechanically elevated from outside of the maze and the mouse was allowed to freely explore the EPM for 30 min. Behavior was video recorded and analyzed using Ethovision v.9 (Noldus, Leesburg, VA, United States). The parameters evaluated in this test are the distance moved and time spent in the open and closed arms of the EPM after 30 min.

### NTG-Induced Hind Paw Mechanical Allodynia

Mechanical thresholds were determined with von Frey monofilaments (VFF; eight filaments, range 0.07–2 g, Stoelting Co, Wood Dale, IL) using a modified up-and-down method (Chaplan et al., 1994; Kaufmann et al., 2016). On each testing day, a maximum of 12 animals were used. Mice were individually confined in clear acrylic cages (22 cm × 22 cm × 12 cm) divided into four chambers, each on a raised wire mesh platform that allowed full access to the tested paws. Mice were acclimated for 2 h, on the day of testing and 1 day prior. Mechanical thresholds were evaluated before (*baseline*), 75, and 120 min after i.p. administration of 10mg/kg NTG (American Regent Inc, Shirley, NY, United States), in accordance with NTG’s time-to-peak-effect in this model (Bates et al., 2010; Kaufmann et al., 2016). Filaments were applied in both ascending (beginning with 0.07 g VFF) and descending (beginning with 2 g VFF) staircase protocols (Kaufmann et al., 2016). Each filament was applied perpendicular to the center of each hind paw five times, spaced 1 s apart. In the absence of a response, the next VFF in the series was applied until a response was witnessed. Response to VFF was recorded as an immediate withdrawal of the tested hind paw to the applied stimulus, with or without an observed licking behavior. The withdrawal threshold was quantified as the mean of ascending and descending threshold values for each paw (Kaufmann et al., 2016). The total mechanical withdrawal threshold was evaluated as the mean of both hind paws. The experimenter performing the test was blinded to the treatment group.

### Cortical Spreading Depression (CSD) Model

The CSD model was performed according to a previously published method (Kaufmann et al., 2016). Due to the duration of the CSD protocol, only two animals were evaluated per day. Each animal was anesthetized with isoflurane (5% induction, 1.1–1.4% maintenance) and mounted on a stereotaxic frame (Kopf Instruments). Throughout the experiment, the animal’s vital signs (oxygen saturation, heart rate, respiratory rate, body temperature) were monitored and stabilized using a physiological monitoring apparatus (PhysioSuite, Kent Scientific). The parietal skull was exposed between bregma and lambda, and the region 0.5 mm posterolateral to bregma, anterolateral to lambda, and medial to the temporal ridge was thinned to transparency. A burrhole was created 0.5 mm from the temporal ridge, midway between bregma and lambda for KCl solution application. The cortex was illuminated by a white-light LED (5500K, Phillips Lumileds) and reflected light (optical intrinsic signal; OIS) was collected with a lens system consisting of two f/0.95 lenses connected front to front focused on a high-sensitivity 12-bit charge-coupled device camera (MiCAM02, Brainvision). Images were acquired at 2 Hz for the duration of the experiment. CSDs were then induced by continuous perfusion of 1M KCl over the burrhole using a syringe pump (1 ml/h) for 2 h. CSD frequency was analyzed immediately after termination of the experiment in a blinded manner (Kaufmann et al., 2016).

### Optical Intrinsic Signal Analysis

All analyses were performed blind as to the treatment group. Ratio images [% (R-R_0_)/R_0_] were generated from each recording, with an average of the first 8 frames serving as R_0_. In each experiment, two circular regions of interest (ROIs, 8 × 8 pixels) were placed 1 and 2 mm medial to the KCl burr hole, perpendicular to the advancing CSD wavefront, in areas devoid of surface vessels. A plot representing the change in cortical reflectance over time was generated for each ROI.

### CSD Evaluation

Cortical spreading depression were identified by multiphasic, concentric changes in OIS (Kaufmann et al., 2016). “*Full*” CSDs were defined as CSDs that propagated concentrically across the whole imaging field; “*Partial*” CSDs, did not propagate across the whole imaging field (Kaufmann et al., 2016). The total CSD count includes both full and partial CSDs, as no difference was observed between the two measures. Velocity of CSD propagation was calculated only for the full CSDs, using the difference in time between peak OIS reflectance at ROIs 1 and 2.

### Statistics

All analysis was performed in a blinded manner, using Graphpad Prism v5.03. A *p* < 0.05 was considered significant. All data was tested for normality using the D’agostino and Pearson omnibus test. All data were distributed normally except for CSD velocity. Except for mechanical allodynia, all data are presented as box whisker plots and results are presented as *median* (*25^th^ percentile*, *75^th^ percentile*). Weights were evaluated using a Two-way ANOVA test matched by groups. In the SI test, we used a Two-way ANOVA test matched by groups and a paired *t*-test with a Bonferroni correction. Comparisons for baseline allodynia were made using the Student’s *t*-test (CVS paradigm) and Tukey multiple comparison test (SDS). Data in the mechanical allodynia tests are presented as mean ± SEM, and were compared using repeated measure ANOVA followed by a Tukey multiple comparison test. Comparison between CSD frequencies were evaluated using the Tukey multiple comparison test (SDS) and Student’s *t*-test (CVS). CSD velocity were not normally distributed and were evaluated using the Kruskal–Wallis test followed by a Dunns’ test (SDS) and Mann Whitney test (CVS).

## Results

### Physiological Verification of Stress Response

**FIGURE 2:**
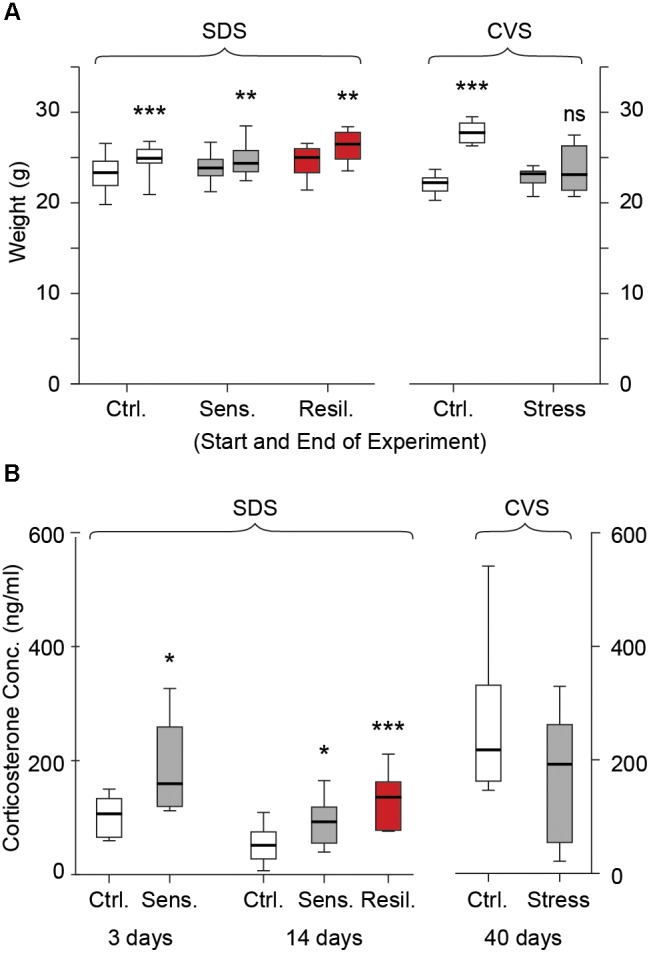
Weight gain and plasma corticosterone levels. **(A)** Box whisker plots show weight gain in the SDS and CVS paradigm. No differences were seen between the groups in the SDS paradigm at the start or end of the paradigm (*p* > 0.05). All groups showed a significant increase in weight gain during the 14 days (*p* < 0.001 for control and *p* < 0.01 for sensitive and resilient groups, Two-way ANOVA, *n* = 14, 12, and 6 for control, stress sensitive and stress resilient groups respectively). No significant difference was seen between the control and stress mice at the beginning of the CVS paradigm. After 40 days, control mice showed a significant increase in weight gain while no change in weight gain was seen for stressed mice (NS *p* > 0.05, ^∗∗∗^*p* < 0.001, Two-way ANOVA, *n* = 12, 11 for the control and stress mice respectively). **(B)** Plasma concentrations on day 3 and 14 of SDS, and day 40 of CVS. Stress mice had increased plasma corticosterone levels compared to controls after 3 days of social defeat (*p* < 0.05, Student’s *t*-test, *n* = 6 per group). Both stress sensitive and stress resilient mice showed a significant increase in plasma corticosterone levels compared to control after 14 days (*p* < 0.05 for sensitive, *p* < 0.001 for resilient groups, Tukey multiple comparison test, *n* = 14, 12, and 6 for control, stress sensitive and stress resilient groups respectively). No significant difference was seen in corticosterone levels after the CVS paradigm between stressed and control mice. (*p* > 0.05, Student’s *t*-test, *n* = 6 per group).

We evaluated plasma corticosterone levels subacutely and chronically in the SDS paradigm and chronically in the CVS paradigm using ELISA. Stress mice in the SDS paradigm showed an increase in plasma corticosterone levels compared to controls after 3 days of social defeat [106.7 ng/ml (*65.7, 133.7*) vs. 159.9 ng/ml (*120, 259.4*) for control and stress respectively, *p* < 0.05, **Figure 2B**]. After 14 days of social defeat both stress sensitive and stress resilient mice showed a significant increase in corticosterone levels compared to controls [51.9 ng/ml (*27.6, 75.1*), 92.8 ng/ml (*55.5, 119*), and 136.4 ng/ml (*78.2, 163*) for control, stress sensitive, and stress resilient groups respectively, *p* < 0.05 for stress sensitive, and *p* < 0.001 for stress resilient, **Figure 2B**]. Consistent with prior work showing normalization of corticosterone levels in long-term stress paradigms (Scheich et al., 2017; Caruso et al., 2018), no difference between the groups was seen in the CVS paradigm on day 40 after the end of the stress paradigm [217.7 ng/ml (*162.7, 331.6*) vs. 192.7 ng/ml (*54.7, 262.5*) for control and stress respectively, *p* > 0.05, **Figure 2B**]. In order to provide an additional biological readout of stress in both stress paradigms, we monitored animal weight. As expected, CVS animals gained weight at a significantly slower rate than control mice (**Figure 2A**). No difference between weights was seen between control and stress mice at the beginning of the CVS paradigm. However, following 40 days, stressed mice showed a significantly reduced weight compared to their control counterparts [22.3 g (*21.3*, *22.7*) vs. 23.2 g (*22.2*, *23.5*) at the beginning of the experiment; 27.8 g (*26.7*, *28.8*) vs. 23.1 (*21.4*, *26.3*) at the end of the experiment, for control and stress mice respectively, *p* < 0.001, **Figure 2A**]. In the SDS paradigms no differences were seen in animal weights at the beginning [23.1 g (21.7, 24.3) vs. 23.6 g (22.8, 24.6) vs. 24.8 g (23.1, 25.7) for control, stress sensitive and resilient respectively, *p* > 0.05, **Figure 2A**] or end [24.7 g (24.2, 25.6) vs. 24.1 g (23.2, 25.5) vs. 26.2 g (24.6, 27.5) for control, stress sensitive and resilient respectively, *p* > 0.05, **Figure 2A**] of the stress paradigm between controls, stress sensitive, or stress resilient mice. All groups showed a significant increase in weight gain at the end of the SDS paradigm compared to day 1 (*p* < 0.001, **Figure 2A**).

### Social Interaction Test

**FIGURE 3:**
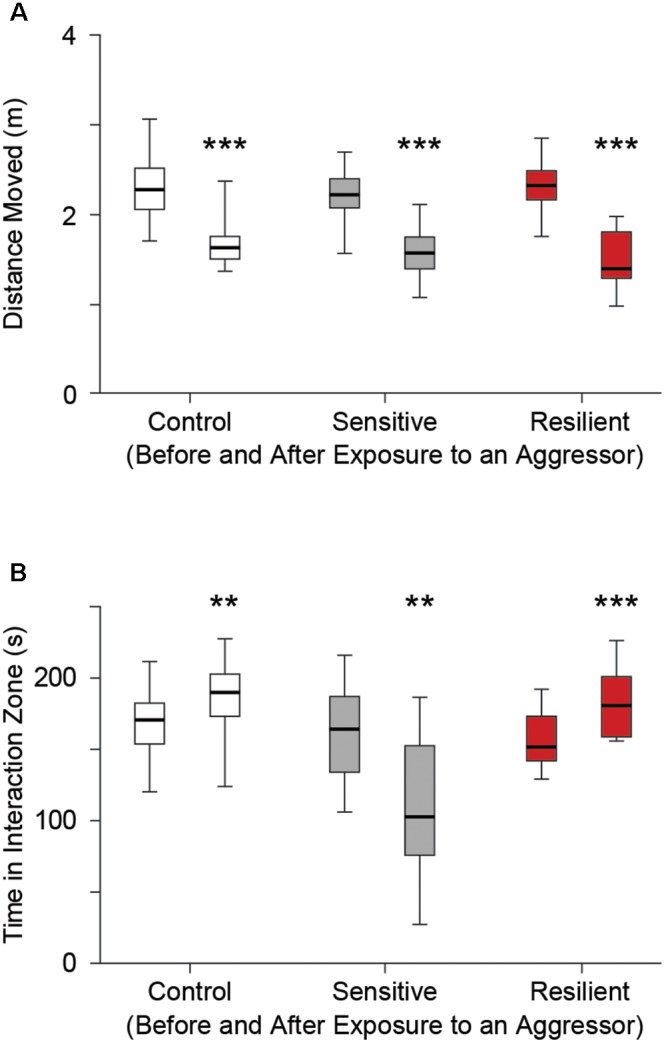
Social interaction test. **(A)** Distance moved for each group before (left box whisker) and after (right box whisker) placement of a novel aggressor mouse (retired CD-1 breeder) in the control, stress sensitive, and stress resilient groups. There was a significant reduction in distance moved in all three groups after placement of an aggressor compared to when an aggressor wasn’t present (^∗∗∗^*p* < 0.001, Two way ANOVA, *n* = 28, 16, 12 for the control, stress sensitive, and stress resilient groups respectively). The similar reduction in distance moved for all groups establishes that differences in social interaction in **(B)** are not due to differences in mobility. **(B)** Time spent in the interaction zone before and after placement of a novel aggressor. Both control and stress resilient mice showed a significant increase in time spent in the interaction zone after placement of the aggressor. Stress sensitive mice showed a significant reduction in the time spent in the interaction zone after placement of a novel aggressor compared to when the aggressor was not present. This shows the effect of social defeat on the stress sensitive mice compared to their control or resilient counterparts (^∗∗^*p* < 0.01, ^∗∗∗^*p* < 0.001, Paired student’s *t*-test with Bonferroni correction, *n* = 28, 16, 12 for the control, stress sensitive and stress resilient groups respectively).

In SDS mice, we evaluated distance moved and time spent in the interaction zone before and after placement of an aggressor. All groups showed a significant reduction in distance moved on the second trial, with an aggressor present [2.3 m (*2, 2.5*) vs. 1.6 m (*1.5, 1.8*); 2.2 m (*2.1, 2.4*) vs. 1.6 m (*1.4, 1.8*); 2.3 m (*2.2, 2.5*) vs. 1.4 m (*1.3, 1.8*) for before vs. after placement of a novel aggressor in the control, stress sensitive, and stress resilient groups respectively, *p* < 0.001, **Figure 3A**]. Stress sensitive mice showed a significant reduction in time spent in the interaction zone while an aggressor was present compared to when the aggressor was not present [164 s (*134, 187*) vs. 103 s (*76, 153*) for before vs. after the aggressor was present, *p* < 0.01, **Figure 3B**]. In contrast, both control and stress resilient mice showed an increase in the time spent in the interaction zone after placement of aggressor compared to before [171 s (*154*, *183*) vs. 190 s (*173*, *203*) *p* < 0.01; 152 s (*142*, *173*) vs. 181 s (*169*, *201*) *p* < 0.001, for before vs. after placement of the aggressor in control and stress resilient mice respectively, **Figure 3B**]. This enabled us to calculate a SI index and separate the mice after social defeat to 16 stress sensitive and 12 stress resilient mice (Golden et al., 2011).

### Behavior Testing

#### Open Field

**FIGURE 4:**
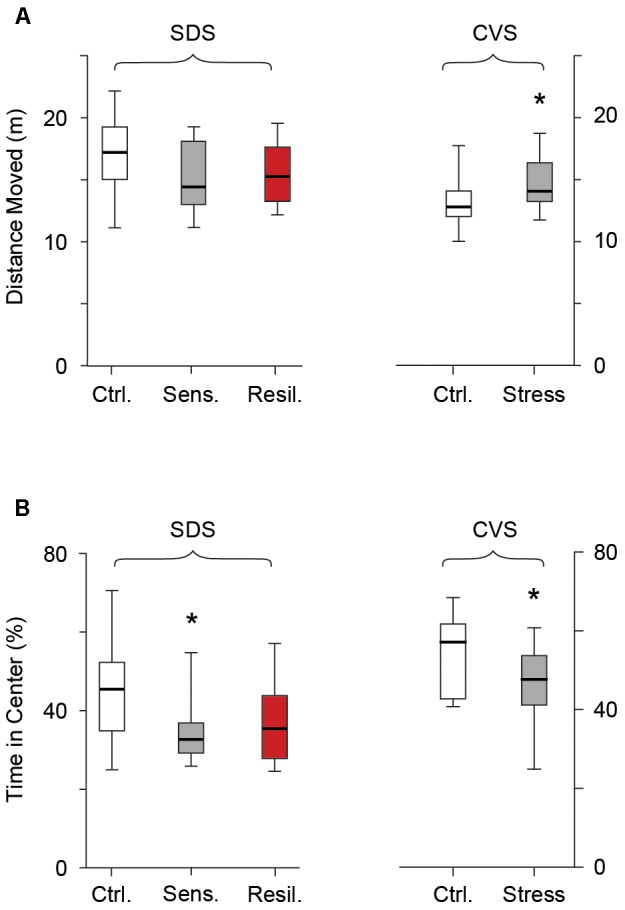
Open field test. Box whisker plots show distance moved **(A)** and time spent in the center **(B)** following the SDS and CVS paradigms. In the SDS paradigm no difference was seen in distance moved between the control, stress sensitive and stress resilient mice. However, stress sensitive, but not stress resilient mice, showed a reduction in time spent in the center of the open field compared to their control counterparts (^∗^*p* < 0.05, Tukey multiple comparison test, *n* = 28, 16, 12 for the control, stress sensitive and stress resilient mice respectively). In the CVS paradigm, despite an increase in total distance moved in chronically stressed mice, there was a significant decrease in time spent in the center of the open field compared to control counterparts (*p* < 0.05, Student’s *t*-test, *n* = 12, 11 for control and stress mice respectively).

We evaluated the distance moved and time spent in the center of the open field arena the day after completion of both SDS and CVS paradigms. In the SDS paradigm no difference in distance moved was seen between the control, stress sensitive, and stress resilient mice [17 m (*15.1, 19.2*), 14.4 m (*13.1, 18.1*), 15.3 m (*13.3, 17.6*) for control, stress sensitive, and stress resilient mice, *p* > 0.05 **Figure 4A**], confirming there was no locomotion difference between groups. However stress sensitive, but not stress resilient mice, spent significantly less time in the center compared to their control counterparts [42.5% (*34.2, 52.3*), 32.7% (*29.3, 36.9*), 35.3% (*27, 42.1*) for control, stress sensitive and stress resilient mice, *p* < 0.05 for stress sensitive vs. control, **Figure 4B**]. No significant difference was seen between stress sensitive and stress resilient mice in time spent in center (*p* > 0.05, **Figure 4B**), however, the values for stress resilient mice were also not significantly different from controls. Interestingly, in the CVS paradigm, chronically stressed mice showed an increase in distance moved compared to their control counterparts [12.7 m (*11.9, 14*) vs. 13.9 m (*13.1, 16.2*) for control and stress respectively, *p* < 0.05, **Figure 4A**]. However they too showed a significant decrease in time spent in the center of the arena compared to controls [57.1% (*42.8, 61.7*) vs. 47.7% (*41.2, 53.7*) for control and stress mice respectively, *p* < 0.05, **Figure 4B**], consistent with a behavioral stress response.

### Elevated Plus Maze

**FIGURE 5:**
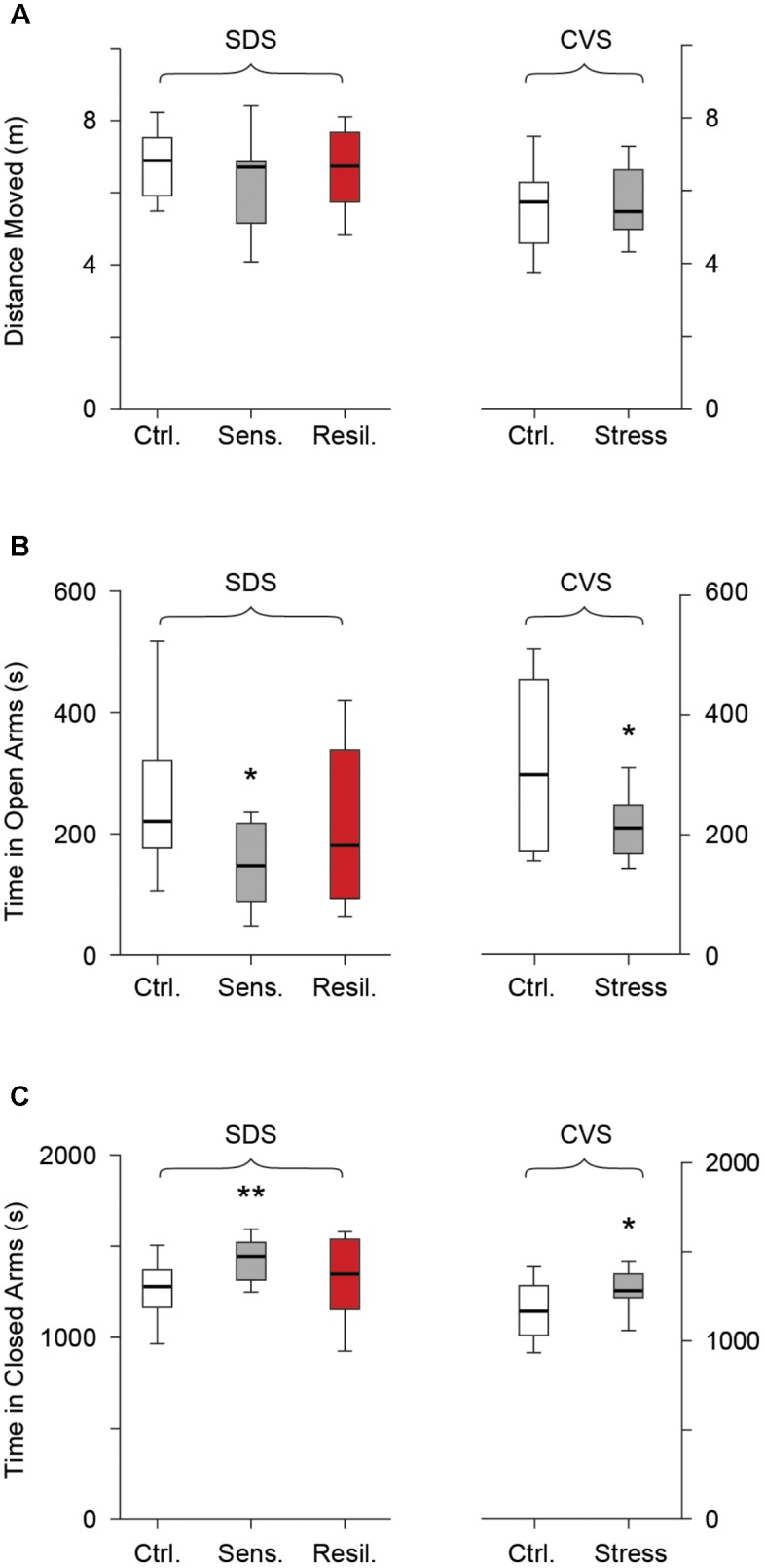
Elevated plus maze. Box whisker plots show SDS paradigm and CVS paradigm results. **(A)** No difference in distance moved in the SDS paradigm (*p* > 0.05, Tukey multiple comparison test, *n* = 28, 16, 12 for control, stress sensitive, and stress resilient groups). No difference in distance moved in the CVS paradigm (*p* > 0.05, Student’s *t*-test, *n* = 12, 11 for control and stress respectively). **(B,C)** Time spent in the open arms and closed arms respectively in both paradigms. Stress sensitive mice in the SDS paradigm spent less time in the open arms and significantly more time in the closed arms compared to controls (^∗^*p* < 0.05, ^∗∗^*p* < 0.01, Tukey multiple comparison test, *n* = 28, 16, 12 for control, stress sensitive and stress resilient respectively). There was a significant decrease in time spent in the open arms and a significant increase in the time spent in the closed arms for the stress mice vs. controls in the CVS paradigms. (^∗^*p* < 0.05, Student’s *t*-test, *n* = 12, 11 for control and stress mice respectively).

We further evaluated behavioral anxiety phenotypes using the EPM on the second day after completion of both stress paradigms. No difference in distance moved was seen between any of the groups in the SDS paradigm [6.8 m (*5.9, 7.5*), 6.7 m (*5.2, 6.9*), and 6.7 (*5.7, 7.7*) for the control, stress sensitive, and stress resilient groups respectively, *p* > 0.05, **Figure 5A**] or between the control and stressed mice in the CVS paradigm [5.6 m (*4.6, 6.2*) vs. 5.4 m (*4.9, 6.5*) for control and stress groups respectively, *p* > 0.05, **Figure 5A**]. Stress sensitive SDS mice spent significantly less time in the open arms [221 s (*176.8, 322*), 148 s (*88.9, 217.4*), and 181 s (*93.8, 338.5*) for the control, stress sensitive and stress resilient groups respectively, *p* < 0.05, **Figure 5B**] and significantly more time in the closed arms [1277 s (*1649, 1348*), 1445 s (*1314, 1519*), and 1347 s (*1153, 1537*) for the control, stress sensitive, and stress resilient groups respectively, *p* < 0.01, **Figure 5C**] compared to controls in the SDS paradigm. No significant difference was seen between stress sensitive and stress resilient mice; however, resilient mice values were closer to (and not significantly different from) those of control mice. In the CVS paradigm, chronically stressed mice showed a significant reduction in time spent in the open arms [300.2 s (*72.6, 459.3*) vs. 211s (*169, 248.7*) for the control and stress groups respectively, *p* < 0.05, **Figure 5B**] and a significant increase in the time spent in the closed arms [1168 s (*1032, 1310*) vs. 1283 (*1244, 1376*) for the control and stress groups respectively, *p* < 0.05, **Figure 5C**].

### NTG Induced Mechanical Allodynia

**FIGURE 6:**
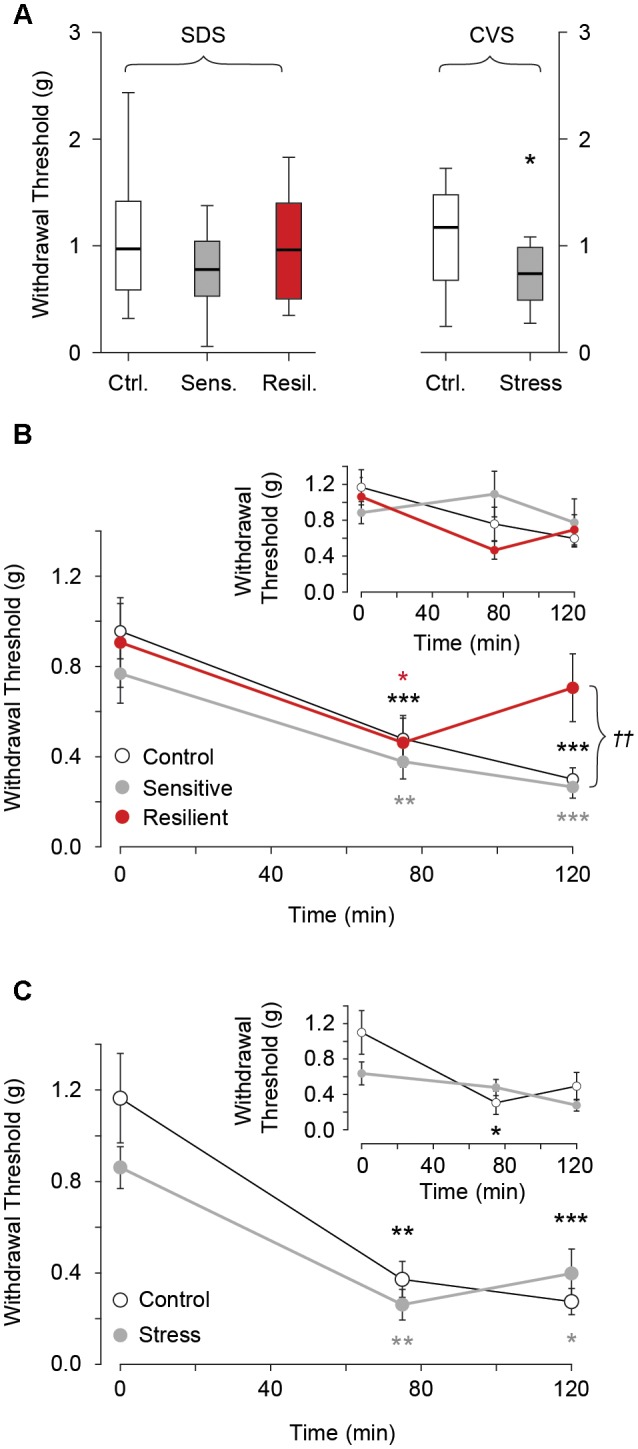
Hind paw mechanical withdrawal threshold before and after i.p. administration of 10 mg/kg NTG or vehicle control (insets). **(A)** Baseline mechanical withdrawal threshold in SDS and CVS paradigms. No differences between the control, stress sensitive, and stress resilient groups in the SDS paradigm (*p* > 0.05, Tukey multiple comparison test, *n* = 28, 16, 12 for the control, sensitive, and resilient groups respectively). In the CVS paradigm there was a significant reduction in baseline mechanical allodynia threshold in chronically stressed mice compared to controls (^∗^*p* < 0.05, Student’s *t*-test, *n* = 12, 11 for the control and stress groups respectively). **(B)** In the SDS paradigm, NTG significantly lowered withdrawal threshold for control and stress sensitive groups at both 75 and 120 min compared to baseline. In contrast, stress resilient mice showed a significant reduction in withdrawal threshold compared to baseline at 75 min but *not* the 120 min (^∗^*p* < 0.05, ^∗∗^*p* < 0.01, ^∗∗∗^*p* < 0.001, Tukey multiple comparison test repeated measures, *n* = 17, 11, 6 for the control, sensitive and resilient groups respectively). The withdrawal threshold at 120 min was significantly higher in resilient compared to control and sensitive groups (^††^*p* < 0.01, Tukey multiple comparison test, *n* = 17, 11, 6 for the control, sensitive and resilient groups respectively). **Inset.** No difference in thresholds after vehicle infusion (*p* > 0.05, Tukey multiple comparison test repeated measures, *n* = 11, 5, 6, for the control, sensitive and resilient groups respectively). **(C)** In the CVS paradigm both control and stress groups showed reduced thresholds compared to baseline, at both time points after NTG. There was no difference between the groups after administration of NTG (^∗^*p* < 0.05, ^∗∗^*p* < 0.01, ^∗∗∗^*p* < 0.001, Tukey multiple comparison test repeated measures, *n* = 6, 5 for control and stress groups respectively). **Inset.** No difference in threshold after vehicle infusion except for the control mice at the 75 min time point. (^∗^*p* < 0.05, Tukey multiple comparison test repeated measures, *n* = 6 per group).

We evaluated hind-paw mechanical withdrawal threshold before and after NTG administration in both SDS and CVS paradigms. There was no difference in withdrawal threshold between the control, stress sensitive, and stress resilient groups in the SDS paradigm (1.04 g ± 0.1, 0.8 g ± 0.1, 0.98 g ± 0.14 for the control, stress sensitive, and stress resilient groups respectively, *p* > 0.05, **Figure 6A**). Following i.p. administration of 10 mg/kg NTG both control and stress sensitive groups showed a significant reduction in hind paw mechanical withdrawal threshold after 75 min (0.96 g ± 0.12 vs. 0.48 ± 0.1 for controls, *p* < 0.001; 0.77 g ± 0.13 vs. 0.37 g ± 0.25 for stress sensitive, *p* < 0.01; for baseline and 75 min NTG respectively, **Figure 6B**) and 120 min time points (0.96 g ± 0.12 vs. 0.3 ± 0.05 for controls; 0.77 g ± 0.13 vs. 0.27 g ± 0.05 for stress sensitive; for baseline and 120 min after NTG respectively, *p* < 0.001, **Figure 6B**). In contrast, resilient mice showed a significant reduction at the 75 min time point (0.9 g ± 0.2 vs. 0.46 g ± 0.11 for baseline and 75 min NTG respectively, *p* < 0.05, **Figure 6B**) but *not* the 120 min time point (0.9 g ± 0.2 vs. 0.7 g ± 0.15 for baseline and 120 min NTG respectively, *p* > 0.05, **Figure 6B**). No significant change in mechanical withdrawal threshold was seen after administration of vehicle control in all three groups (*p* > 0.05, **Figure 6B** inset), implying that the reductions in threshold we observed were due to NTG.

In the CVS paradigm, chronically stressed mice showed a significant reduction in hind paw withdrawal threshold at *baseline* compared to their control counterparts (1.13 g ± 0.14 vs. 0.74 g ± 0.09 for control and stress groups respectively, *p* < 0.05, **Figure 6A**). Both groups showed a significant reduction in mechanical withdrawal threshold after administration of NTG at the 75 min (1.17 g ± 0.2 vs. 0.37 g ± 0.08 control, *p* < 0.01; 0.86 g ± 0.09 vs. 0.26 g ± 0.07 chronic stress, for baseline and 75 min NTG, *p* < 0.01, **Figure 6C**) and 120 min time point (1.17g ± 0.2 vs. 0.27 g ± 0.06 control, *p* < 0.001; 0.86 g ± 0.09 vs. 0.4 g ± 0.1 chronic stress, *p* < 0.05, for baseline and 120 min NTG, **Figure 6C**) compared to baseline. However no difference *between* the groups was seen during these time points following administration of NTG. Following administration of vehicle no difference in mechanical allodynia was seen compared to baseline in both groups except for control mice at the 75 min time point (1.1 g ± 0.25 vs. 0.3 g ± 0.13 for baseline and 75 min vehicle, *p* < 0.05, **Figure 6C** inset).

### Cortical Spreading Depression Model

**FIGURE 7:**
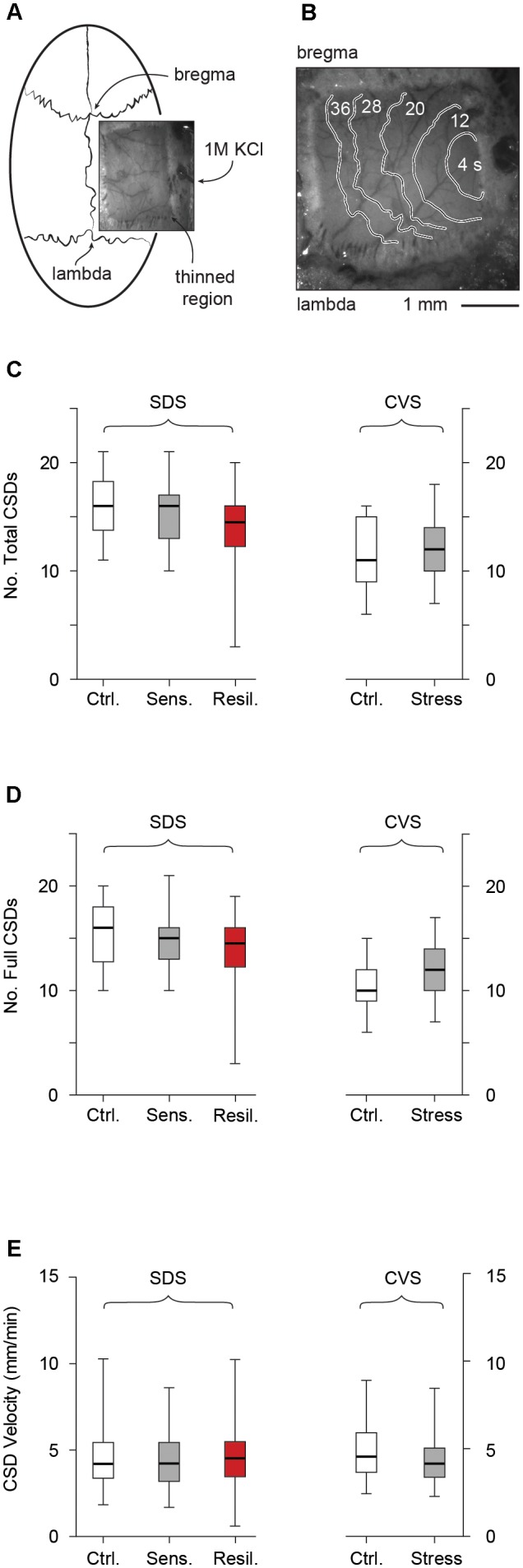
Cortical spreading depression. **(A)** Schematic of CSD experiments, including thin skull preparation between bregma and lambda and location of KCl ejection. **(B)** Optical imaging of the thin skull preparation. CSD wave front is shown by contours labeled by time from initiation of CSD. **(C)** Total number of CSDs (full + partial) after 2 h KCl administration in SDS and CVS paradigms. No difference in CSD frequency was observed (*p* > 0.05, Tukey multiple comparison test, *n* = 28, 16, 12 for control, stress sensitive and stress resilient mice in the SDS paradigm, and student’s *t*-test, *n* = 11 per group in the CVS paradigm). **(D)** Number of full CSDs (excluding partial events) recorded after 2 h KCl administration in both the SDS and CVS paradigms. No difference in CSD frequency was observed (*p* > 0.05, Tukey multiple comparison test, *n* = 28, 16, 12 for control, stress sensitive and stress resilient mice in the SDS paradigm, and student’s *t*-test, *n* = 11 per group in the CVS paradigm). **(E)** CSD velocity in both the SDS (G) and CVS (H) paradigms. No difference in CSD velocity was observed (*p* > 0.05, Kruskal Wallis test followed by Dunn’s test, *n* = 184, 108, 114 events from *n* = 18, 11, 12 mice for the control, stress and resilient groups respectively in the SDS paradigm, and Mann–Whitney test, *n* = 56, 97 events from *n* = 11 mice per group).

The schematic representation of the CSD experiment including optical imaging of the CSD wave front is depicted in **Figures 7A,B**. We evaluated CSD frequency and velocity after completion of the stress paradigm. No CSDs were observed prior to initiation of KCl administration in any of the groups. We evaluated both total (full + partial) and full (omitting partial) CSDs (see methods). In the SDS paradigm, no difference in either total [16 (*13.75, 18.25*), 16 (*13.5, 17*), and 14.5 (*12.25, 16*) for control, stress sensitive, and stress resilient groups respectively, *p* > 0.05, **Figure 7C**] or full [16 (*12.8, 18*), 15 (*13, 16*), and 14.5 (*12.25, 16*) for control, stress sensitive and stress resilient groups respectively, *p* > 0.05, **Figure 7D**] CSD number was observed between control, stress sensitive, and stress resilient mice. Similarly, in the CVS paradigm, we did not detect differences in total [11 (*9, 15*) vs. 12 (*10, 14*) for control and stress groups respectively, *p* > 0.05, **Figure 7C**] or full [10 (*9, 12*) vs. 12 (*10, 14*) for control and stress groups respectively, *p* > 0.05, **Figure 7D**] CSD frequency. Finally, there was no significant difference in velocity between the different groups in either the SDS [4.2 mm/min (*3.4, 5.4*), 4.2 mm/min (*3.2, 5.4*), and 4.5 mm/min (*3.5, 5.5*) for control, stress sensitive and stress resilient groups respectively, *p* > 0.05, **Figure 7E**] or CVS paradigms [4.5 mm/min (*3.7, 5.9*) and 4.2 (*3.4, 5.1*) for control and stress groups respectively, *p* > 0.05, **Figure 7E**].

## Discussion

The complex effects of stress on general nociception are widely studied in both clinical and preclinical models (McEwen, 1998; Blackburn-Munro and Blackburn-Munro, 2001; Chapman et al., 2008; Johnson and Greenwood-Van Meerveld, 2014; Ortego et al., 2016; Burke et al., 2017). Stress is also widely reported as both a trigger of individual migraine attacks, and a factor in migraine chronification (Chabriat et al., 1999; Wober et al., 2006; Rains, 2009; Borsook et al., 2012; May and Schulte, 2016). However, there has been comparatively less preclinical work to examine the effects of stress on migraine models. We evaluated the effects of chronic stress on migraine relevant phenotypes using two different paradigms commonly used in stress research.

### Induction of Stress Phenotypes

Mice experiencing social defeat showed an increase in plasma corticosterone concentrations compared to their control counterparts after both 3 and 14 days of social defeat. This result was similar to other published reports using social defeat paradigms (Li et al., 2014; Sial et al., 2016; Niraula et al., 2018). No differences were seen between the stress sensitive and resilient groups, and both had a significantly higher plasma corticosterone concentrations compared to controls. In the CVS paradigm, however, we did not detect a difference in corticosterone concentration between stress and control mice. This is likely because on chronic timescales the corticosterone response is heterogeneous, with some studies showing an increase (Harpaz et al., 2013), and others, like ours, showing no change in serum levels (Scheich et al., 2017; Caruso et al., 2018). As an alternative measure (Willner, 2017), we recorded weight changes and observed less weight gain, consistent with prior work (Gamaro et al., 2003). No differences in weight gain were seen in the SDS paradigm between any of the tested groups. A previous work evaluated weight gain using the social defeat paradigm and showed a significant difference in weight gain between control and stress groups after 19 days but not before (Tramullas et al., 2012). This might indicate that expected weight changes after stress may be seen after more prolonged time periods. A different study showed an increase in weight gain for social defeat mice compared to controls (Goto et al., 2014). However, their paradigm included 0.5 min of defeat time compared to 5 min employed by our protocol. Since we used food deprivation in our CVS paradigm, one might suspect that the weight gain changes observed are due to our use of food deprivation and not the stress paradigm. However, we further measured the animals’ weights on the day of CSD test (the animals had food and water *ad libitum* for 7 days after termination of the stress paradigm), to indicate a significantly reduced weight for the stress mice compared to controls (data not shown). In our hands, 4 days is sufficient for mice to gain significant weight, and therefore we think that our observed weight gain changes in this paradigm are due to stress and not food deprivation *per se*. Overall, for both paradigms we observed physiological responses consistent with a stress response.

We further evaluated the validity of both stress models by their ability to produce anxiety behaviors in the open field and EPM (Handley and McBlane, 1993; Holmes et al., 2000; Lipkind et al., 2004; Carobrez and Bertoglio, 2005). In the open field test, we found that both stress sensitive males in the SDS paradigm and chronically stressed males in the CVS paradigm spent significantly less time in the center. This was not due to a decrease in locomotion in either case. Similarly, in the EPM, in both stress paradigms mice showed a reduction in the time spent in the open arms and an increase in the time spent in the closed arms compared to controls that was not due to locomotion decrease. The combined results in both behavioral tests indicate that both paradigms successfully produced an anxiety related behavioral phenotype.

### Effects of Stress on Migraine Models

Having successfully established physiological and behavioral stress responses, we evaluated the effects of the stress paradigms on assays that have face validity in preclinical migraine research. In humans with migraine but not control subjects, NTG causes a delayed migraine (without aura) indistinguishable from spontaneous attacks (Iversen, 1995; Tassorelli et al., 1999). NTG testing is thus used as a preclinical model for migraine without aura (Bates et al., 2010; Greco et al., 2014; Pradhan et al., 2014; Kaufmann et al., 2016; Moye and Pradhan, 2017). CSD is the physiological substrate of the migraine aura in humans, and thus is used in preclinical models of migraine with aura (Ayata et al., 2006; Chen et al., 2006; Brennan et al., 2007; Theriot et al., 2012). As stress affects the whole organism (de Kloet et al., 2005; Sapolsky, 2015) and can cause excitability and plasticity changes at all levels of the neuraxis (Blackburn-Munro and Blackburn-Munro, 2001; Wellman, 2001; Vyas et al., 2002; Bains et al., 2015; McEwen, 2016; McEwen et al., 2016), we reasoned that both a cortical-excitability model (CSD) and a more nociception-based model (NTG) could be affected.

### Increased Baseline Mechanical Allodynia in CVS

In the NTG test, we observed a significant reduction in baseline mechanical threshold (before NTG administration) in chronically stressed CVS mice, but not in stress sensitive SDS mice. In the pain literature, both SDS and CVS can induce heterogeneous responses. SDS has been reported to produce no alterations in algesic response (Li et al., 2014; Liu et al., 2015), hypoalgesia (Rodgers and Shepherd, 1989; Tramullas et al., 2012), and hyperalgesia (Sawicki et al., 2018). Similarly CVS has produced no difference (Liu et al., 2017), hypoalgesia (Shi et al., 2010) and hyperalgesia (Gamaro et al., 2014; Lomazzo et al., 2015). These results are consistent with a larger literature on the algesic effects of stress (Jennings et al., 2014); in general chronic stress alone appears insufficient to determine the direction of the algesic response, which results from a combination of parameters including the type and duration of each stressor, the length of the complete stress paradigm and the type of animal and the specific animal strain used.

We interpret the CVS response in our hands as evidence for a *generalized* algesic response to stress, as the reduction in withdrawal threshold *preceded* administration of a putatively migraine-specific agent (NTG). The implication is that there may be a non-specific favoring of algesic processes by CVS that might have an impact on multiple pain conditions (including migraine), albeit not in a migraine-specific manner. As for the difference between SDS and CVS results, we suspect these differences reflect differences in both the modality (psychosocial vs. multimodal) and duration (14 vs. 40 days) of the two stress paradigms.

### NTG-Induced Mechanical Allodynia Does Not Differ Between Stress and Control Groups

In both SDS and CVS paradigms, NTG induced a significant reduction in mechanical withdrawal threshold, observed both 75 and 120 min after NTG challenge. This was likely due to NTG and not to algesic effects of the injection itself, as with one exception (control mice in the CVS paradigm at the 75 min time point) administration of vehicle did not reduce mechanical withdrawal threshold. Though all groups exhibited decreases in withdrawal thresholds consistent with mechanical allodynia, we observed no differences *between* the groups after administration of NTG. We interpret this as evidence that social defeat and CVS do not exert *migraine-specific* algesic effects. Given the generalized reduction in withdrawal thresholds in CVS treated mice and the high dose of NTG used (10 mg/kg), it is possible that our data are subject to a “floor effect,” limiting the dynamic range of the NTG test and potentially generating a false negative result. That said, we evaluated mechanical withdrawal threshold up to 120 min post NTG administration. Mechanical withdrawal threshold was shown to return to pre-NTG baseline values 4 h after administration of NTG (Bates et al., 2010). It is possible that control mice may display a different recovery profile from NTG compared to stressed mice at these later time points. This point remains to be tested. Overall, we conservatively interpret the NTG data in CVS as consistent with a *non-migraine-specific* algesic response. This kind of response has potential clinical relevance as it could be expected to affect both migraine and the comorbid pain conditions that so frequently accompany migraine (Burstein et al., 2000a,b; Goadsby et al., 2017). Furthermore, chronic intermittent administration of NTG to mice has been used to model progression of migraine from an acute to a chronic state (Pradhan et al., 2014). Chronic stress was reported to exacerbate the progression of a headache disorder from episodic to chronic condition (Nash and Thebarge, 2006; Rains, 2009). Therefore a future study might evaluate the effects of our stress paradigms on the development of mechanical allodynia after chronic intermittent administration of NTG.

### Possible Resistance to NTG Effects in Stress-Resilient Mice

Interestingly, resilient mice in the SDS paradigm showed an attenuated mechanical withdrawal response after NTG that was not seen with controls or stress sensitive mice. Indeed, we have not observed such a resistance to high dose (10 mg/kg) NTG in any other experimental group unless the animals were treated with anti-migraine drugs (Bates et al., 2010; Brennan et al., 2013; Pradhan et al., 2014; Kaufmann et al., 2016; Ben Aissa et al., 2017). There is some indication from the pain literature that stress resilience is associated with alterations in pain phenotypes: stress resilient rodents show increased pain thresholds and a resistance to stress induced hyperalgesia, that in some cases correlate with a reduced neuroendocrine stress response (Alvarez et al., 2015; Alvarez et al., 2018; Genty et al., 2018). Similarly, stress resilience in humans is associated with reduced symptom burden in several pain states, including migraine (Ong et al., 2010; Sturgeon and Zautra, 2010; McAllister et al., 2015). We interpret these data as consistent with a potentially *migraine-specific* resilience-induced resistance to pain.

### No Stress Effect on CSD Susceptibility

We observed no differences in CSD number or velocity between stressed or control mice in either the SDS or CVS paradigms. This is unlikely to be related to our ability to induce stress, since we observed physiological and behavioral evidence of stress in both paradigms. Our results are consistent with those of Shyti et al. (2015) who found that neither 20 min nor 3 h restraint stress altered CSD susceptibility. Our results expand on this data by showing that even more severe 14- and 40-day stress paradigms do not alter CSD susceptibility in wild type animals. However, it is important to note that *neither* set of results rules stress out as a modulator of CSD – indeed in mice carrying the FHM1 familial hemiplegic migraine mutation (but not wild type littermates), 20 mg/kg corticosterone (but not either form of restraint stress) was associated with increased CSD susceptibility (Shyti et al., 2015). Thus it remains possible that more severe stress paradigms in mice carrying migraine mutations could alter CSD phenotypes. Moreover, we and Shyti et al. (2015) tested only male mice; both wild type and migraine mutant female mice have an increased susceptibility to CSD (Brennan et al., 2007; Eikermann-Haerter et al., 2009). It is possible that stress effects, which are also sexually dimorphic (McEwen and Milner, 2017), affect females differently with regard to CSD susceptibility. Overall, while it might at first appear implausible to suggest that a massive depolarization like CSD could be affected by stress, such a conclusion might be hasty: stress has the ability to alter cortical synaptic strength and plasticity (Robert et al., 2013; Bains et al., 2015), and thus potentially increase excitability, in a manner analogous to synaptic migraine mutations (Tottene et al., 2009; Dilekoz et al., 2015; Brennan and Pietrobon, 2018). Furthermore, we chose to look at CSD frequency and velocity instead of CSD threshold, as a measure of CSD susceptibility. We were previously successful in utilizing this method to evaluate the efficacy of a novel anti-migraine compound (Kaufmann et al., 2016), and the effects of sex differences on migraine relevant mutations (Brennan et al., 2007) on CSD susceptibility. Interestingly, a recent work evaluated the effects of acute and chronic stress on CSD threshold in swiss albino mice. Similar to our work, this work showed that both acute and chronic stress did not change CSD frequency but did reduce CSD threshold (Yapici-Eser et al., 2018). Even though our study used different mice and stress paradigms, it is possible that stressed mice might show a difference in CSD threshold but not frequency compared to controls. Clearly more work is needed on this subject.

## Conclusion

In this investigation of the effects of stress on migraine-relevant phenotypes in male mice, we show that CVS reduces baseline algesic thresholds in a non-specific manner that may be relevant not only to migraine but to comorbid pain conditions. We also show that mice resilient to SDS show resistance to a migraine-specific pain stimulus. We found no effect of stress on susceptibility to CSD in our paradigms. This work contributes to the systematic preclinical examination of stress effects on migraine models.

## Author Contributions

DK and KB conceived and designed these experiments, analyzed and interpreted the data, and wrote the manuscript. DK performed the experiments and wrote the first draft of this manuscript.

## Conflict of Interest Statement

The authors declare that the research was conducted in the absence of any commercial or financial relationships that could be construed as a potential conflict of interest.
