# Supplementary material for: The Effects of Chronic Stress on Migraine Relevant Phenotypes in Male Mice
Source: Front Cell Neurosci. 2018 Sep 19;12:294. doi: 10.3389/fncel.2018.00294 (PMC6156251; doi:10.3389/fncel.2018.00294)
Supplement: Supplementary file 1 [file Table_1.DOCX]

Supplementary Material

**The effects of chronic stress on migraine relevant phenotypes in male mice.**

Dan Kaufmann^1^, K.C.Brennan^1^*

***** Corresponding Author:

K.C. Brennan

University of Utah,

Department of Neurology,

383 Colorow Drive, Room 364

Salt Lake City, Utah 84108

USA.

Tel. +1 801 581 8129

Fax. +1 801 585 0625

E-mail. [K.C.Brennan@hsc.utah.edu](mailto:K.C.Brennan@hsc.utah.edu) (K.C. Brennan)

| **Day** | **Morning session** | **Afternoon session** |
| --- | --- | --- |
| 1 | 5 min rat encounter |  |
| 2 | 5 min predator odor | 5 min rat encounter |
| 3 | 30 min restrained stress | 10 min tail suspension |
| 4 | 5 min predator odor | 5 min rat encounter |
| 5 | 10 min tail suspension |  |
| 6 | 5 min rat encounter | 24h food + water deprivation |
| 7 | Continued food and water deprivation |  |
| 8 | 5 min predator odor + 30 min restrained stress | 5 min rat encounter |
| 9 | 10 min tail suspension | 24h wet bedding |
| 10 | continued wet bedding | 5 min rat encounter |
| 11 | 10 min tail suspension | 24 h cage tilt |
| 12 | continued cage tilt | 24 h wet bedding |
| 13 | continued wet bedding |  |
| 14 | 30 min restrained stress |  |
| 15 | 10 min tail suspension | 10 min rat encounter |
| 16 | 30 min restrained stress | 24 h food + water deprivation |
| 17 | continued food + water deprivation | 24h cage tilt |
| 18 | continued cage tilt | 10 min tail suspension |
| 19 | 5 min rat encounter + 24 h wet bedding + food + water deprivation |  |
| 20 | continued wet bedding and water deprivation | 10 min tail suspension |
| 21 | 5 min rat encounter |  |
| 22 | 1h restrained stress | 5 min predator odor |
| 23 | 24 h food + water deprivation | 5 min rat encounter |
| 24 | **No stress** |  |
| 25 | 5 min predator odor + 30 min restrained stress | 10 min rat encounter |
| 26 | 24h food + water deprivation + wet bedding |  |
| 27 | end of wet cage + food and water deprivation |  |
| 28 | 10 min rat encounter |  |
| 29 | 30 min restrained stress | 15 min tail suspension |
| 30 | 24 h cage tilt |  |
| 31 | 5 min rat encounter |  |
| 32 | 30 min restrained stress | 15 min tail suspension |
| 33 | **No stress** |  |
| 34 | 24h food + water deprivation + wet bedding |  |
| 35 | 30 min restrained stress |  |
| 36 | 5 min tail suspension in rat cage | 24 h cage tilt |
| 37 | 5 min tail suspension in rat cage |  |
| 38 | 24h food + water deprivation + wet bedding |  |
| 39 | 5 min tail suspension in rat cage |  |
| 40 | 10 min rat encounter |  |

**Supplementary Table 1.** Protocol for 40 days of chronic variable stress. The stressors were employed twice daily (morning and afternoon sessions) and included the following: rat encounter, predator odor, restrained stress, tail suspension, wet bedding, food and water deprivation, and cage tilt. The stressors were employed in a random manner to prevent habituation.
